# Ezetimibe reduces cholesterol content and NF-kappaB activation in liver but not in intestinal tissue in guinea pigs

**DOI:** 10.1186/s12950-017-0150-y

**Published:** 2017-02-02

**Authors:** Peter Fraunberger, Elisabeth Gröne, Hermann-Josef Gröne, Heinz Drexel, Autar K. Walli

**Affiliations:** 1 Medical Central Laboratories, Carinagasse 41, A-6800 Feldkirch, Austria; 2 Department of Cellular and Molecular Pathology, German Cancer Research Center, Heidelberg, Germany; 3 Vorarlberger Institute of Vascular Investigation and Treatment (VIVIT), Dornbirn, Austria; 4 Institute of Laboratory Medicine, Ludwig-Maximilians-University, Munich, Germany

**Keywords:** Inflammation, Cholesterol, Ezetimibe, Guinea pigs, Liver, Intestine

## Abstract

**Background:**

Statins (HMG CoA reductase inhibitors), in addition to reducing circulating cholesterol and incidence of coronary heart disease, also have pleiotropic, anti-inflammatory effects. Patients with chronic liver diseases, non-alcoholic fatty liver disease (NAFLD) or hepatitis C are often excluded from statin therapy because of adverse effects in a small cohort of patients despite increased cardiovascular risk cholesterol. Ezetimibe, which inhibits cholesterol absorption by inhibition of Niemann-Pick C1 like 1 (NPC1L1) protein in the brush border of intestinal cells, has been suggested as a new therapeutic option in these patients.

**Methods:**

Effects of ezetimibe on lipoprotein metabolism, hepatic and intestinal lipid content in guinea pigs, an animal model with a lipoprotein profile and pattern similar to humans were investigated. In order to investigate a possible effect of ezetimibe on cholesterol induced inflammation NF-kappaB activation as an indicator for inflammatory processes in liver and gut tissue was measured.

**Results:**

Lipid enriched diet led to accumulation of lipids in hepatic tissue which caused strong hepatic NF-kappaB activation. Ezetimibe reduced lipid diet induced increase of circulating cholesterol by about 77% and prevent hepatic NF-kappaB activation almost completely. In contrast in intestinal cells Ezetimibe, though lowering diet induced cholesterol accumulation, increased triglyceride content and subsequent NF-kappaB activation.

**Conclusion:**

In summary these data show, that ezetimibe effectively reduced diet induced circulating cholesterol levels, hepatic lipid accumulation and inflammatory response in our guinea pig model. However this drug elicited a local inflammatory response in intestinal tissue. Whether these diverse effects of ezetimibe on inflammatory parameters such as NF-kappaB have clinical relevance remains to be determined.

## Background

Although statins (HMG CoA reductase inhibitors) very rarely cause clinically significant liver injury, asymptomatic elevation in aminotransferases is common [1]. Therefore patients with chronic liver diseases, non-alcoholic fatty liver disease (NAFLD) or hepatitis C are often excluded from statin therapy despite increased cardiovascular risk [2]. To achieve a relevant reduction of cholesterol in plasma, cholesterol absorption inhibitor ezetimibe [1-(4-fluorophenyl)-(3R)-[3-(4-fluorophenyl)-(3S)-hydroxypropyl]-(4S)-(4-hydroxypropyl)-2-azetidinone] has been suggested as an additional therapy to low dose statins. Combination of low dose statins (10 mg/day) with administration has even been shown to be more effective than statin monotherapy [3, 4]. Recent data show that ezetimibe also reduces insulin resistance, dyslipidaemia and hepatic fat accumulation in patients with NAFLD [5–7]. Therefore this drug has been suggested as a new therapeutic option in these patients. Hepatic fat accumulation is an essential feature of NAFLD and may stem from dietary fat, from adipocytes via lipolysis and from de novo lipogenesis. Although fatty liver is thought to be benign, accumulation of lipids can lead to lipotoxicity resulting in inflammation, probably due to oxidative processes [8, 9]. Specifically lipotoxicity can induce cell proliferation, NF-kappaB activation, release of proinflammatory cytokines and subsequent cell death. Although the extent of inflammation varies considerably in steatohepatitis, in some cases it may lead to hepatic injury, cirrhosis and hepatocellular carcinoma [10].

Ezetimibe inhibits cholesterol absorption as well as its reuptake via the enterohepatic cycle in the mucosa of the small intestine without affecting the absorption of triglycerides or fat-soluble vitamins [11]. The molecular target of ezetimibe is the Niemann-Pick C1 like 1 (NPC1L1) protein in the brush border of intestinal cells although other proteins may be also involved [12]. NPC1L1 protein is necessary to transport unesterified cholesterol from the intestine to the endoplasmatic reticulum, where it is esterified and assembled into chylomicrons particles. Inhibition of NPC1L1 protein thereby decreases the amount of cholesterol delivered by chylomicrons to the liver [13]. However NPC1L1 receptor is also highly expressed in hepatocytes and is thought to play a role in regulating biliary cholesterol concentration [14]. This may explain recent observations, that inhibition of NPC1L1 by ezetimibe modulates hepatic and metabolic disorders in rodents [15–17]. However value of studies in animal models such as mice and rat is limited because LDL is not the main cholesterol transporting lipoprotein and hepatic lipid metabolism may differ from humans. Ezetimibe reduces LDL by about 18–20% which may be effective reduction in borderline patients, but unfortunately no detailed studies about clinical safety of this drug are available to date [18].

Statins, which effectively reduce circulating cholesterol, have been shown to exhibit a wide variety of immunomodulatory effects independent of their lipid lowering effects [19]. In contrast, pleiotropic effects of ezetimibe are still under debate. Whereas some studies indicate an anti-inflammatory effect [20, 21], other studies found no effects on inflammation [22, 23].

Accordingly this study was designed to determine the effects of ezetimibe on lipid metabolism, tissue lipid content and NF-kappaB activation in hepatic and intestinal tissue of guinea pigs. This animal model was chosen because its lipoprotein profile, response to statins, hepatic HMG-CoA reductase activity, and rates of hepatic cholesterol synthesis are similar to humans [24].

## Methods

### Biochemicals

All chemicals and biochemicals were obtained from Sigma Chemie GmbH (München, FRG), E. Merck GmbH (Darmstadt, FRG) or Roche (Mannheim, FRG). Bovine serum albumin was obtained from Behring (Marburg, FRG). Fetal calf serum was from Gibco Europe Ltd. (Eggstein, FRG). ^125^I-sodium iodide, DL[2-^3^H]-mevalonic acid and ^14^C-cholesteryloleate were purchased from Amersham Buchler GmbH & Co. KG (Braunschweig, FRG), 3-hydroxyl[3-^14^C]-methylglutaryl CoA (HMG-CoA) from Du Pont, NEN Products (Boston, MA, USA). Ezetimibe [1-(4-fluorophenyl)-(3R)-[3-(4-fluorophenyl)-(3S)-hydroxypropyl]-(4S)-(4-hydroxypropyl)-2-azetidinone] was provided by Merck, Sharp & Dome Resarch Laboratories (West Point, PA, USA).

### Animal procedures

Male Dunkin-Heartley guinea pigs (300–400 g body weight) obtained from Charles River, Kisslegg, Germany, were maintained in a light-cycle room (dark from 7:00 a.m. to 7:00 p.m., light from 7:00 p.m. to 7:00 a.m.) and had free access to food and water ad libitum. Diets were prepared as previously described by Conde et al. [25]. All diets were prepared and pelleted by Fa. ssniff G® (Germany). Composition of chow diet was as follows: 21% protein, 3% fat, 14% fiber, 8,3% essential minerals, 0,007% vitamin E and 0.015% vitamin C. Cholesterol diet contained 0,2% cholesterol and 15% fat consisting olive, palm kernel and safflower oil (1:2:1,8). Ezetimibe diets were prepared as described above but contained in addition 0.005% ezetimibe.

After 1 week of adaptation guinea pigs were maintained on the indicated diets for further 2 weeks. Animal groups were as follow: Chow diet (*n* = 12), Cholesterol diet (*n* = 12), Chow diet plus Ezetimibe (*n* = 4), Cholesterol diet plus Ezetimibe (*n* = 8). All experiments were carried out between 8 and 10 am.

### Blood and tissue samples

Blood was obtained by aortic puncture and collected in tubes containing EDTA. These tubes were centrifuged at 5,000 g for 15 min at 4 °C. Plasma was separated and stored at 4 °C. Liver tissue was frozen by Wollenberger technique in liquid N_2_ [26] and stored at −196 °C for biochemical analysis. Gut tissue was washed twice with saline. Liver and gut tissue were frozen immediately in liquid nitrogen (−180 °C) for immunohistological studies.

### Clinical chemistry measures

Cholesterol and triglyceride plasma levels [mg/dl] as well as aspartate aminotransferase (AST), alanine transaminase (ALT) and gamma-glutamyl transpeptidase (gGT) activities in plasma [U/l] were measured with Roche automated system (Integra 800, Roche diagnostics).

### Electrophoretic mobility shift assay (EMSA)

Nuclear extracts from gut and liver tissue: Nuclear extracts were prepared as described by Fukuma et al. [27]. Briefly, tissue was homogenized in homogenizing buffer (10 mM HEPES-buffer pH 7.6, containing 0.1 mM EDTA, 15 mM KCl, 2 mM MgCl_2_, 1 mM DTT, 1 mM phenylmethylsulfonyl fluoride (PMSF) and 1% protease inhibitor cocktail). This homogenate was centrifuged at 850xg and supernatant was discarded. Cell pellet was incubated in homogenizing buffer containing 0.2% Igepal (Sigma, Germany) on ice for 10 min and centrifuged. After discarding the supernatant pellet was rinsed in homogenizing buffer containing 0.25 sucrose, centrifuged for 5 min at 850 × g and supernatant was discarded. Finally pellet was suspended in nuclear extraction buffer (50 mM HEPES, pH 7.9, containing 0.1 mM EDTA, 0.4 mM KCl, 10% glycerol, 1 mM DTT, 0.5 mM PMSF and 1% protease inhibitor cocktail) and was shaken for 30 min at 4 °C, centrifuged at 12,000 × g for 5 min. Supernatant (nuclear extract) was kept frozen at −80 °C.

DNA binding assay: NF-kappaB consensus oligonucleotides GPNFKB-01 L: 5′-AGT-TGA-GGG-GAC-TTT-CCC-AGG-C-3′ and GPNFKB-02 k: 5′-GCC-TGG-GAA-AGT-CCC-CTC-3′ (Eurogentec, Belgien) were annealed and labeled with [alpha-^32^P]dATP (Amersham Germany) in the presence of deoxynucleoside triphosphates by primer extension with the Klenow fragment of DNA polymerase I (Roche, Mannheim, Germany). Nuclear proteins were incubated with ^32^P labeled oligonucleotide in binding buffer (10 mM Tris-buffer pH 7.5, containing 50 mM NaCl, 5 mM MgCl_2_, 1 mM EDTA, 10% glycerol, 5 μg bovine serum albumin, 0.2% Nonidet P-40, 4 μg poly-(dI-dC) und 1 mM DTT) for 15–30 min. DNA bound NF-kappaB was separated by electrophoresis on 5% acryl/bisacrylamid gel in 0.25× Tris borate EDTA buffer (4 °C). Each lane contained 5 μg nuclear protein and 60,000 CPM. Gels were dried, exposed to storage phosphor screens, read in a phosphorimager (Storm, Molecular Dynamics, USA) and quantified using ImageQuant software (ImageQuant®, Molecular Dynamics, USA).

### HMG CoA reductase activity in liver microsomes and LDL binding to hepatic membranes

Isolation of liver microsomes and incubation of microsomes with [^14^C]-HMG-CoA was performed as described by Walli and Seidel [28], with the exception that [^14^C]-mevalonolactone was separated from ^14^C-HMG-CoA by column chromatography. Lactonised incubates (0.05 ml) were applied to a 0.5 × 5 cm column containing 100–200 mesh Bio-Rex, chloride form (Bio Rad, Munich, FRG) which was equilibrated with distilled water. [^14^C]-mevalonolactone was eluted with 2 ml of distilled water directly into scintillation vials. After addition of 10 ml Ultima Gold scintillation fluid (Packard Instr., Zürich, Switzerland) radioactivity was measured in a scintillation counter. Binding of LDL to hepatic membranes was assayed according to Kovanan et al. [29]. Briefly membranes were isolated from homogenized liver tissue by ultracentrifugation. For binding assay ^125^I labeled apo-E rich human VLDL was used as a ligand. Maximal binding affinity was calculated by scatchard plot analysis.

### Lipid content in liver and gut

Tissue lipids were measured according to Carr et al. [30]. ^14^C-cholesteryloleate was added as an internal standard.

### Histology

Liver and intestine tissue were removed from animals under deep anesthesia, quickly blotted free of blood, weighed, and processed for histology, immunohistology, and gel shift analysis. Tissue samples, 3–4 μm in thickness, were prepared using cryostat technology (CM1900, Leica), mounted on poly-L-lysine coated slides, dried at room temperature for 1 h and stained with oil red for fat staining. For immunohistology samples were cut into 1-mm thick slices, immersed in 4% formaldehyde and then embedded in paraffin. In addition tissue slices were snap frozen in liquid nitrogen and stored at −80 °C for further analysis.

### Immunohistology

Immunohistochemical staining was done on sections of paraffin-embedded tissue samples. Primary antibody mouse anti-human NF-kappaB p50 (Santa Cruz Biotechnology, USA) was diluted 1:200 with PBS containing 0.05% Tween and 1% BSA. Biotinylated rabbit anti-mouse immunoglobulins (DAKO, Germany), diluted 1:200 in PBS-BSA served as a secondary antibody. After 30 min incubation, slides were washed twice in PBS-Tween for 10 min each. This was followed by incubation with streptavidin-horse reddish peroxidase (HRP) (DAKO, Germany) diluted 1:200 for 20 min. Substrate 3-amino-9-ethyl -carbazole (ACE) (0.01%) containing 0.006% H_2_O_2_ was added shortly before use for colour development. Sections were counterstained with Harris modified Hematoxylin 1:4 (Sigma, Germany) for 30 s. Slides were examined under light microscope and film EPY 64 T (Kodak, Germany) was used for photographs.

**Fig. 1:**
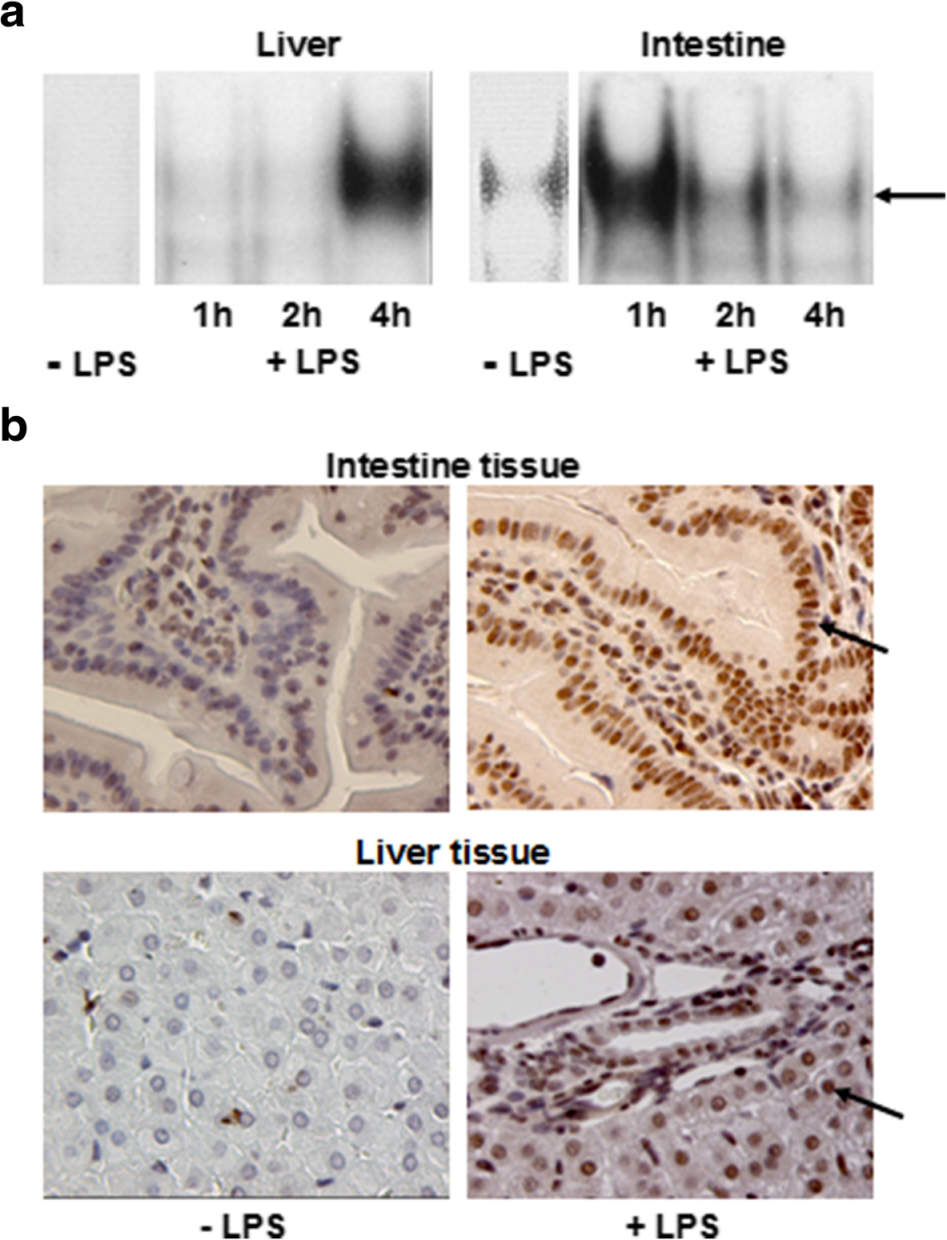
Representative gelshift assays of NF-kappaB in nuclear extracts from (**a**) and immuncytochemical detection of NF-kappB (**b**) in liver and intestine to establish suitability of gel shift assay and immunohistology in this model. Animals were treated with saline or LPS (2 mg/kg body weight) and killed after the indicated time to obtain liver and intestine tissue. Arrow denotes nuclear NF-kappaB staining

In order to establish, whether gel shift assay as well as immunohistology was suitable in the present model, guinea pigs were treated intraperitoneally with LPS (2 mg/kg body weight) to induce endotoxinemia and sepsis. Figure 1 shows gelshift analysis of NF-kappaB activation in liver and gut tissue by EMSA (A) and immunohistology (B). As expected, LPS activated NF-kappaB in both, liver and intestine tissue.

### Statistics

All data are given as means ± standard deviation (SD) and compared using the Wilcoxon, Mann-Whitney rank sum test for non-parametric data and t-test for parametric data. *P* values < 0.05 were considered as statistically significant.

## Results

### Effect of ezetimibe on lipoprotein metabolism

Chow diet together with ezetimibe decreased circulating levels of cholesterol in guinea pigs by 42% whereas triglyceride levels remained unchanged. Cholesterol diet increased these levels by ten and three fold, respectively compared to chow diet. Cholesterol diet in combination with ezetimibe decreased circulating cholesterol by about 77% compared to cholesterol diet alone without altering triglyceride levels (Table 1).

**Table 1:** Plasma levels of cholesterol and triglycerides in guinea pigs on chow or cholesterol diet with or without Ezetimibe

Diet	n	Cholesterol [mg/dl]			Triglycerides [mg/dl]		
Chow	11	36	±	18	114	±	60
Ezetimibe	4	21	±	5^a^	72	±	5
Cholesterol	12	303	±	91^b^	302	±	168^b^
Cholesterol + Ezetimibe	7	69	±	28^c^	281	±	105^b^

^a^significantly different from Chow-diet, ^b^significantly different from Chow and Ezetimibe diet, ^c^significantly different from Ezetimibe and Cholesterol diet

Ezetimibe diet increased HMG-CoA reductase activity by about 2,6 fold whereas cholesterol diet inhibited enzyme activity almost completely (Table 2). However cholesterol enriched diet together with ezetimibe prevent this effect. Maximal binding of apo-E rich VLDL by isolated hepatic membranes was unaffected by ezetimibe. Cholesterol enriched diet increased hepatic membrane binding by about 30%. Cholesterol diet in combination with ezetimibe did not significantly affect maximal binding capacity of apo-E rich ligand on hepatic membranes. In contrast a minimal reduction of binding affinity as reflected by Km in animals on chow or cholesterol enriched diet was noted after ezetimibe treatment.

**Table 2:** HMG-CoA reductase activity in liver microsomes, Vmax and Km in liver membranes in guinea pigs on chow or cholesterol diet with or without Ezetimibe

Diet	n	HMG-CoA reductase [pmol/mg Protein/min]	Vmax [μg / mg Protein]	Km [μg / ml Medium]
Chow	11	22,2 ± 7,2	1,22 ± 0,21	23,6 ± 4,05
Ezetimibe	4	57,0 ± 28,7^a^	1,39 ± 0,11	21,56 ± 1,8
Cholesterol	12	1,2 ± 0,5^b^	1,8 ± 0,45	35,50 ± 7,5
Cholesterol + Ezetimibe	7	25,4 ± 7,0^c^	1,76 ± 0,35	32,24 ± 7,0

^a^significantly different from Chow-diet, ^b^significantly different from Chow and Ezetimibe diet, ^c^significantly different from Ezetimibe and Cholesterol diet

### Effect of ezetimibe on liver enzyme activities in plasma

Cholesterol feeding induced a significant increase of markers of liver disease such as aspartate aminotransferase (AST), alanine transaminase (ALT) and gamma-glutamyl transpeptidase (gGT). In particular ALT, which is recognized as a biochemical marker for fatty liver disease, was increased about 2 fold. All three parameters returned to normal circulating values on cholesterol enriched diet containing Ezetimibe. Ezetimibe alone had no effect on these markers (Table 3).

**Table 3:** Circulating levels of AST, ALT and y-GT in guinea pigs on chow or cholesterol diet with or without Ezetimibe

Diet	n	AST [U/l]	ALT [U/l]	gGT [U/l]
Chow	12	56 ± 13	40 ± 8	7 ± 2
Ezetimibe	4	64 ± 35	41 ± 17	5 ± 1
Cholesterol	12	143 ± 47^a^	69 ± 28^a^	30 ± 27^a^
Cholesterol + Ezetimibe	8	71 ± 16	47 ± 6	10 ± 3

^a^significantly different from Chow diet and Ezetimibe diet

### Effect of ezetimibe on lipid content of liver and intestinal tissue

#### Liver

Ezetimibe did not change hepatic content of cholesterol, triglycerides or phospholipids in animals on chow diet. In animals fed with cholesterol enriched diet hepatic cholesterol, triglycerides and phospholipids increased by 5.7, 8.4 and 1.4 fold, respectively. This increase in cholesterol and phospholipid content was almost completely prevented by addition of Ezetimibe. However triglyceride content in liver tissue was still 2 fold higher when compared to animals on chow diet (Table 4).

**Table 4:** Lipid in content in liver in guinea pigs on chow or cholesterol diet with or without Ezetimibe

Diet	n	Cholesterol [mg/g]	Triglycerides [mg/g]	Phospholipids [mg/g]
Chow	10	1,23 ± 0,3	1,65 ± 0,4	9,56 ± 1,7
Ezetimibe	3	1,32 ± 0,2	1,59 ± 0,1	10,38 ± 0,1
Cholesterol	8	7,22 ± 2,1^a^	13,77 ± 3,1^a^	13,12 ± 3,1^a^
Cholesterol + Ezetimibe	7	1,53 ± 0,3	3,85 ± 1,1^b^	9,73 ± 1,4

^a^significantly different from Chow-, Ezetimibe- and Cholesterol + Ezetimibe diet, ^b^significantly different from chow, Ezetimibe and cholesterol diet

#### Intestine

In animals maintained on chow diet containing ezetimibe no significant changes in cholesterol and phospholipids content but a 2-fold increase in triglycerides was observed. Cholesterol diet increased cholesterol, triglyceride and phospholipid content by 1.54, 3.9 and 1.3 fold, respectively. Cholesterol diet supplemented with ezetimibe reduced cholesterol content by 24% but increased triglyceride content by about 45% fold. Phospholipid content remained unchanged by this diet (Table 5).

**Table 5:** Lipid content in gut in guinea pigs on chow or cholesterol diet with or without Ezetimibe

Diet	n	Cholesterol [mg/g]	Triglycerides [mg/g]	Phospholipids [mg/g]
Chow	11	1,18 ± 0,2	1,69 ± 0,8	5,29 ± 0,9
Ezetimibe	3	0,96 ± 0,3	3,07 ± 1,1^c^	4,29 ± 1,1
Cholesterol	12	1,82 ± 0,3^a^	6,63 ± 2,3^a^	6,65 ± 1,9
Cholesterol + Ezetimibe	6	1,39 ± 0,3^d^	9,67 ± 2,5^b^	6,97 ± 1,3

^a^significantly different from Chow, Ezetimibe and Cholesterol + Ezetimibe diet, ^b^significantly different from Chow, Ezetimibe and Cholesterol diet, ^c^significantly different from Chow, Cholesterol and Cholesterol + Ezetimibe diet, ^d^significantly different from Chow and Cholesteroldiet

**Fig. 2:**
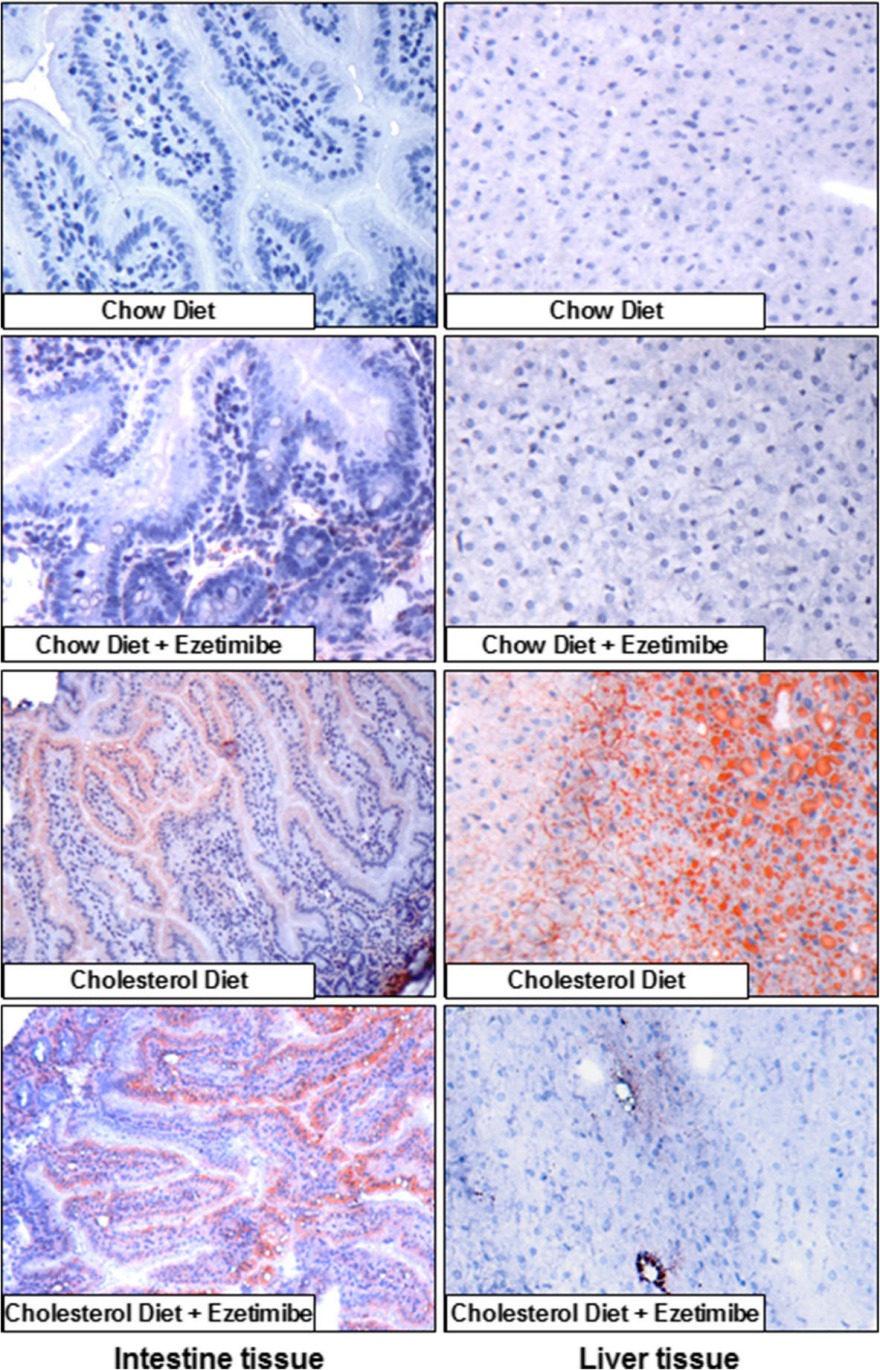
Oil red staining of liver and intestine tissue of guinea pigs maintained on various diets. Animals were fed with the indicated diet for 2 weeks. Oil red staining was performed on frozen liver and intestine sections

Next we stained frozen sections of hepatic and intestinal tissue for neutral lipids (Fig. 2). Cholesterol enriched diet led to increased neutral lipid staining in both hepatic and intestinal tissue. Animals maintained on cholesterol enriched diet containing ezetimibe showed decreased neutral lipid staining only in hepatic tissue whereas in intestinal tissue no decrease of neutral lipids was noted.

### Effect of ezetimibe on NF-kappaB activation

#### Liver

**Fig. 3:**
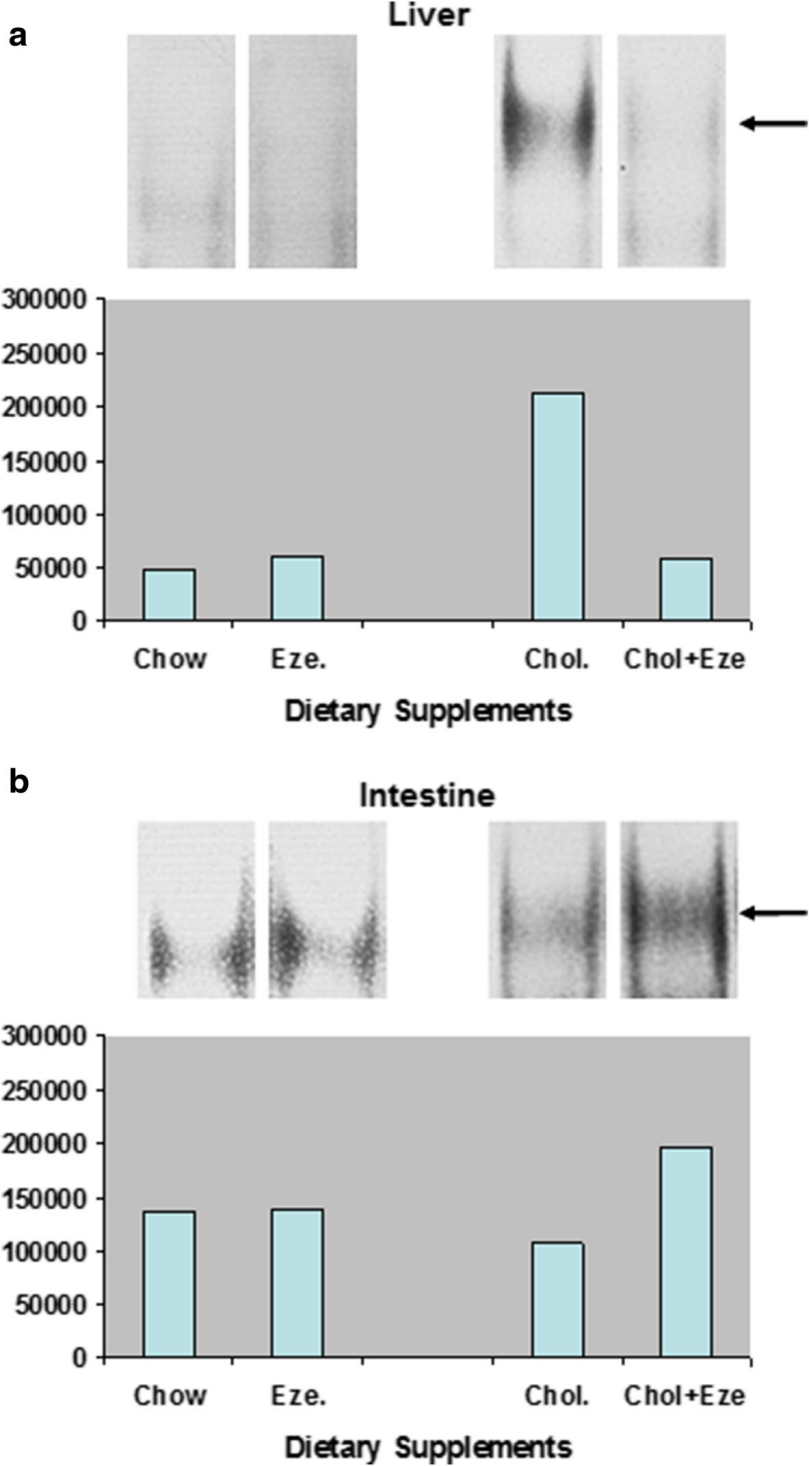
Representative gelshift assays of NF-kappaB in nuclear extracts from liver (**a**) and intestine (**b**). Guinea pigs were maintained on the indicated diets for 2 weeks. Gel shift assays were performed on frozen liver and intestine tissue. Arrow denotes nuclear NF-kappaB band. Quantification of individual bands was done by ImageQuant software (ImageQuant®, Molecular Dynamics, USA)

Gel shift assays in nuclear extracts from liver tissue showed about 4 fold activation of NF-kappaB by a cholesterol enriched diet (48,337 ± 14,558 vs. 213,650 ± 79,521 arbitrary units, *p* < 0.05). Addition of ezetimibe to cholesterol enriched diet almost completely abolished this NF-kappaB activation (213,650 ± 79,521 vs. 58,555 ± 25,843 arbitrary units, *p* < 0.05). Ezetimibe without cholesterol diet had no effect on hepatic NF-kappaB activation (Fig. 3a).

#### Intestine

Ezetimibe alone did not alter NF-kappaB activation (Fig 3b). However in animals fed on cholesterol diet, addition of ezetimibe induced approximately 2 fold increase of NF-kappaB activation.

**Fig. 4:**
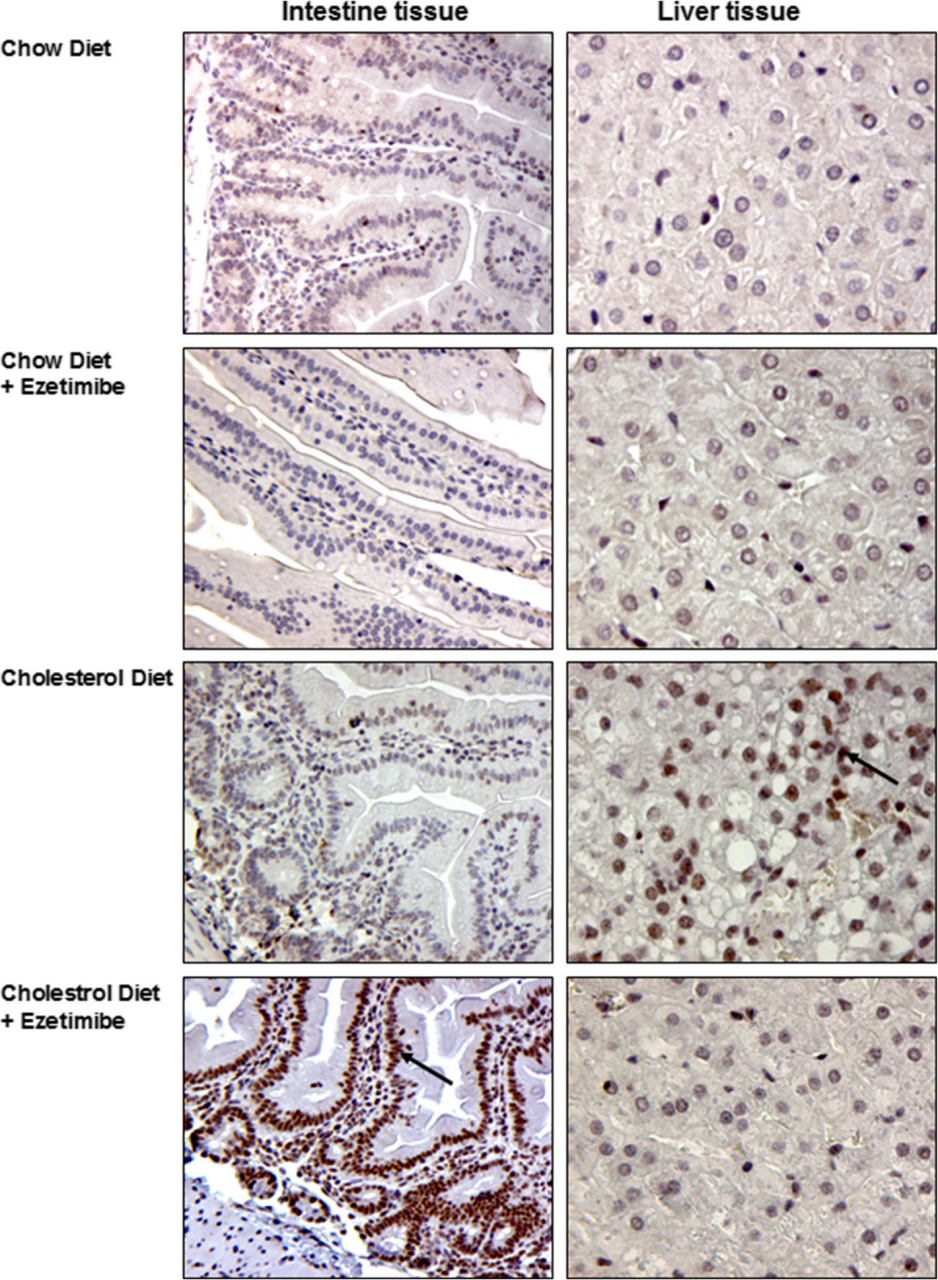
Representative micrograph of Immuncytochemical detection of NF-kappB. Guinea pigs were maintained on the indicated diets for 2 weeks. Immunhistology was performed on paraffin fixed liver and intestine sections. Arrow denotes nuclear NF-kappaB staining

This ezetimibe induced NF-kappaB activation was confirmed by immunohistological studies (Fig. 4). In animals on cholesterol diet an increased staining of NF-kappaB in nuclei of liver cells could be detected, whereas addition of ezetimibe to cholesterol diet prevented this effect. In animals fed with ezetimibe alone, no nuclear staining of NF-kappaB was visible. In intestine tissue neither cholesterol nor ezetimibe alone induced NF-kappaB activation. However when ezetimibe was added to cholesterol diet a strong increase of NF-kappaB staining was noted in nuclei of intestinal cells.

## Discussion

Ezetimibe is a new lipid-lowering agent that inhibits intestinal absorption of dietary and biliary cholesterol with subsequent decrease of about 10–20% in circulating cholesterol levels both in human and animal studies. However, it has not been shown, whether monotherapy with ezetimibe has any beneficial effect in CHD patients [18]. Although combination of low dose statins (10 mg/day) with ezetimibe administration lowers circulating LDL-cholesterol, combination of statins with niacin is superior to ezetimibe in lowering carotid intima thickness [31]. Furthermore some studies raised concerns about a higher cancer incidence in patients treated with ezetimibe plus simvastatin [32].

We therefore investigated effects of ezetimibe on guinea pigs, which are a suitable animal model because their lipoprotein profile, rates of cholesterol synthesis and dietary response is similar to humans. It has been shown, that combinations of high dietary cholesterol with increased fat content effectively increase circulating LDL cholesterol in this animal model. Furthermore cholesterol lowering drugs such as statins strongly reduce the dietary induced LDL cholesterol levels [24, 25].

In our study we found a 77% reduction of high cholesterol diet induced circulating cholesterol with Ezetimibe. Furthermore hepatic HMG-CoA reductase activity, which was strongly suppressed by cholesterol diet, returned to control level in ezetimibe treated animals which indirectly confirms the cholesterol lowering effect of the drug in this animal model. It is well established that enterocytes derive their cholesterol by absorption from the intestinal lumen, intracellular synthesis and uptake of LDL from the plasma. Under physiological conditions the amount of absorbed cholesterol by far exceeds new synthesis as well as receptor mediated uptake from the plasma. Enterocytes takes up dietary cholesterol and also cholesterol from enterohepatic recirculation. In the present study cholesterol enriched diet increased cholesterol and triglyceride content of intestine by 1,5 and 3.9 fold, respectively (see Fig. 2 and Table 4).

Ezetimibe inhibits NPC1L1 protein resulting in reduction of micellar cholesterol to the endoplasmatic reticulum which may cause alteration in lipid distribution in chylomicrons [12]. High fat diet loads the intestinal lumen with micellar triglycerides; after entry into enterocytes these are converted to triglyceride rich chylomicrons. During ezetimibe treatment chylomicron particles are cholesterol depleted. Whether their transport out of enterocytes was hampered leading to an intracellular triglyceride rise remains to be analyzed. In a rare genetic disease, chylomicrons retention disease, it has been shown, that assembly of chylomicrons and their delivery into the circulation may be responsible for their retention in intestine. These patients accumulate chylomicrons like lipid droplets containing apoB-48 resulting in a phenotype similar to the histological findings in this study.

In monkeys as well as in humans it has been shown, that treatment with ezetimibe reduce cholesterol content of chylomicrons but did not significantly reduce apo B48 concentrations or kinetics suggesting that ezetimibe reduce LDL cholesterol by decreasing delivery of cholesterol from the intestine to the liver rather than by reducing chylomicrons particle number [33, 34]. These effects may have significance in humans, who usually receive ezetimibe in combination with statins.

Various studies show that ezetimibe does not affect triglyceride absorption; only minor decreases of circulating triglyceride levels have been reported [35, 36]. In our guinea pig model we found an increased intestinal triglyceride content probably due to chylomicron retention but a decrease of circulating triglycerides of by ezetimibe (about 37%) in animals on chow diet, which however was not statistically significant. This decrease though not significant may be due to reduced chylomicrons remnant cholesterol delivery to the liver [13].

In animals on cholesterol enriched diet we found a strong decrease of circulation cholesterol by ezetimibe but statistically insignificant changes of circulating triglycerides. These effects may be due to constant influx of triglyceride rich but cholesterol depleted apoB-48 rich chylomicrons remnants to the liver which result in reduced VLDL production and subsequent reduced circulating LDL, which is the main cholesterol carrying lipoprotein in guinea pigs.

Recent reports suggest a potential benefit of ezetimibe on non-alcoholic fatty liver disease (NAFLD) by improving hepatic insulin sensitivity and decreasing lipid accumulation and hepatic inflammation [37]. Guinea pigs maintained on fat enriched diet mimic human NAFLD. Ezetimibe ameliorates the neutral fat accumulation in these animals as well as markers of liver damage. Cellular lipid accumulation may cause lipotoxicity leading to inflammatory processes and activation of NF-kappaB and subsequent release of inflammatory cytokines such as TNF-a, TGF-b, IL-6 and IL-8 [8, 38–41]. Consistent with this hypothesis activation of NF-kappaB was observed in animals on cholesterol rich diet in our study. However in animals receiving this diet in combination with ezetimibe this activation was prevented. In contrast to liver tissue, neutral fat accumulation as shown by oil red staining (see Fig. 2) and triglyceride content in intestinal tissue was further increased by ezetimibe (392% versus 572% over control (see Table 5). Although triglycerides themselves are probably inert, their hydrolysis to free fatty acids and diacylglycerol may induce inflammatory response. It has been shown that these intracellular fatty acid metabolites lead to endoplasmic reticulum (ER) stress and the activation of NF-kappa B signaling pathways [42, 43]. In our study ezetimibe treatment increase hepatic triglyceride content associated with a significant NF-kappaB activation. Whether this observation is a direct effect of ezetimibe or due to the triglyceride accumulation which may have inflammatory potential cannot be answered by this study.

## Conclusion

The data presented in the present study lead us to conclude, that ezetimibe effectively reduced diet induced circulating cholesterol levels, hepatic lipid accumulation and inflammatory response in our guinea pig model. In contrast this drug elicited a local inflammatory response in intestinal tissue. Whether these diverse effects of ezetimibe on inflammatory parameters such as NF-kappaB have clinical relevance remains to be determined.

## Abbreviations

ALT: Alanine transaminase
AST: Aspartate aminotransferase
CHD: Coronary heart disease
EMSA: Electrophoretic mobility shift assay
gGT: Gamma-glutamyl transpeptidase
NAFLD: Non-alcoholic fatty liver disease
